# Respiratory Disease Surveillance in the Middle East and Latin America during the COVID-19 Pandemic, 2020–2022

**DOI:** 10.3201/eid3014.240303

**Published:** 2024-11

**Authors:** Yeny O. Tinoco, Tamer S. Osman, Julia S. Ampuero, Mahmoud Gazo, Victor Ocaña, Edward Chávez, Marianela Ore, Elizabeth Carrillo, Jose Santa Cruz, Carlos Delgado, Carlos Alvarez, Rommell Gonzalez, Marina S. Gonzalez, Doris Gómez, Maria E. Arango, Javier Jaramillo, Juan M. Pascale, Nicolas Aguayo, Daniel Olson, Kareen Arias, Miguel M. Cabada, William D. Graham, Tyler D. Moeller, Mohammad Alhawarat, Moutasium Hossinate, Fatima Thneibat, Mohammad Maayeh, Bassem A. Hamdy, Omar Nowar, Samuel Y. Levin, Mayar M. Said

**Affiliations:** US Naval Medical Research Unit SOUTH, Callao, Peru (Y.O. Tinoco, J.S. Ampuero, W.D. Graham, T.D. Moeller); US Naval Medical Research Unit EURAFCENT, Cairo, Egypt (T.S. Osman, B.A. Hamdy, O. Nowar, S.Y. Levin, M.M. Said); Jordan Ministry of Health, Amman, Jordan (M. Gazo, M. Alhawarat, M. Hossinate, F. Thneibat, M. Maayeh); Pachitea Health Center, Piura, Peru (V. Ocaña); Centro de Salud Militar, Trujillo, Peru (E. Chávez); Epidemiología, Comando de Salud del Ejército, Lima, Peru (M. Ore); Seguro Social de Salud-EsSalud, Lima (E. Carrillo); Dirección Regional de Salud, Cusco, Peru (J. Santa Cruz); Dirección Regional de Salud, Puerto Maldonado, Peru (C. Delgado); Dirección Regional de Salud Loreto, Loreto, Peru (C. Alvarez); Dirección Regional de Salud, Tumbes, Peru (R. Gonzalez); Secretaria de Salud del Meta, Villavicencio, Colombia (M.S. Gonzalez); Universidad de Cartagena Grupo UNIMOL, Cartagena, Colombia (D. Gómez); Universidad de Antioquia, Medellin, Colombia (M.E. Arango); Hospital Pablo Tobon Uribe, Medellin (J. Jaramillo); Gorgas Memorial Institute of Health Studies, Panama City, Panama (J.M. Pascale); Rayos de Sol NGO, Asunción, Paraguay (N. Aguayo); University of Colorado School of Medicine, Aurora, Colorado, USA (D. Olson); Fundacion para la Salud Integral de los Guatemaltecos, Retalhuleu, Guatemala (K. Arias); Universidad Peruana Cayetano Heredia, Cusco (M.M. Cabada); University of Texas Medical Branch, Galveston, Texas, USA (M.M. Cabada)

**Keywords:** respiratory infections, influenza, COVID-19, SARS-CoV-2, severe acute respiratory syndrome coronavirus 2, viruses, zoonoses, surveillance, Middle East, Latin America

## Abstract

Characterizing the epidemiology of circulating respiratory pathogens during the COVID-19 pandemic could clarify the burden of acute respiratory infections and monitor outbreaks of public health and military relevance. The US Department of Defense supported 2 regions for influenza-like illness and severe acute respiratory infections surveillance, one in the Middle East through US Naval Medical Research Unit EURAFCENT, and another in Latin America through US Naval Medical Research Unit SOUTH. During 2020‒2022, coinciding with the COVID-19 pandemic, we collected a total of 16,146 nasopharyngeal and oropharyngeal swab samples from sentinel sites in Jordan (n = 11,305) and Latin America (n = 4,841). Samples were tested for SARS-CoV-2, influenza, and other respiratory pathogens. SARS-CoV-2 was the most frequently detected pathogen during 2020; other respiratory pathogens had distinct temporal and frequency distributions according to geographic location. Our findings support the need for continued sentinel surveillance as a vital tool for assessing the burden of respiratory diseases globally.

Acute respiratory infection (ARI) surveillance is a tool to monitor shifts in the occurrence and burden of respiratory infections in a population. Since the 2009 influenza pandemic, the World Health Organization (WHO) has recommended implementation of 2 sentinel surveillance programs: the influenza-like illness (ILI) and the severe acute respiratory infection (SARI) programs (*1*). Such programs allow countries and public health authorities to monitor circulating respiratory pathogens, and to understand the seasonality and trends of pathogens, especially those of pandemic potential, such as influenza and coronaviruses (*2*).

Since 2020, the COVID-19 pandemic and public health implementation of preventive measures have had broad effects on persons, communities, and governments. Given that preventive measures were designed to mitigate respiratory virus transmission, notable disruptions to the typical seasonal circulation patterns of common respiratory virus infections, including infections caused by influenza viruses, respiratory syncytial virus (RSV), and others, have been reported globally (*3*).

We describe the changing temporal and geographic pattern of respiratory pathogens in 2 regions using respiratory surveillance programs through the US Naval Medical Research Unit (NAMRU EURAFCENT) in the Middle East and US Naval Medical Research Unit SOUTH (NAMRU SOUTH) in Latin America. Jordan was selected as a representative of the Middle East region because it was the only country among the region that was able to sustain its SARI and ILI surveillance during the pandemic despite all the challenges the healthcare system faced during the study period.

The institutional review board of the US Naval Medical Research Command approved protocol nos. NAMRU3.2007.0003, N3 703, and NAMRU3.PJT.20.0001. The institutional review board of NAMRU SOUTH approved protocol nos. NMRCD.2010.0010, NAMRU6.2011.0012, NAMRU6.2012.0011, NAMRU6.2012.0012, and NAMRU6.2018.0003, in compliance with all applicable federal regulations governing the protection of human subjects.

## Materials and Methods

### Surveillance Sites

The Jordanian Ministry of Health (JMoH) determined ILI and SARI surveillance programs in Jordan as national public health surveillance activities under JMoH responsibility. NAMRU EURAFCENT initiated a collaboration with the JMoH in ILI surveillance activities in 2006 and SARI surveillance in 2010. JMoH supervised all sentinel sites, including SARI (n = 4) and ILI (n = 4) sites. Enrollment was limited to civilian populations who met ILI or SARI case definitions. Surveillance sites in Latin America were in Panama (n = 1), Guatemala (n = 1), Honduras (n = 1), Colombia (n = 7), Peru (n = 22), and Paraguay (*1*) (Appendix Table 1). For the 6 countries, ILI and SARI surveillance activities were conducted in hospitals and clinics where local and US military and civilian populations received medical care. 

In Jordan, after the first COVID-19 case was reported in March 2020, SARI surveillance continued to enroll hospitalized cases as hospitals continued to provide medical care. ILI activities were paused because primary health centers closed during the country lockdown; activities resumed in October 2020. All NAMRU SOUTH surveillance activities in Latin America stopped in March 2020 because of the lockdowns and were resumed progressively, beginning in Honduras in June 2020 and the remaining countries in November 2020.

### Case Definition and Specimen Collection

Respiratory specimens were collected from patients of all ages (except patients <31 days of age in Jordan) who met the ILI or SARI case definition at any of the surveillance sites. SARI and ILI shared the same clinical manifestation; for SARI cases, the need for hospitalization was an additional indicator for severity. JMoH, in collaboration with NAMRU EURAFCENT, used the WHO ILI and SARI case definitions (*4*). NAMRU SOUTH in Latin America used modified WHO ILI and SARI case definitions and a specific case definition for COVID-19 that did not consider fever as a requirement for specimen collection (*5*) (Appendix Table 2). The 2 regions had similar enrollment procedures in which each eligible case was issued a unique study code that was used to link the clinical and epidemiologic information with laboratory data.

Respiratory samples were collected in viral transport medium using nasopharyngeal and oropharyngeal swabs (*4**,**6*). In Jordan, samples were shipped once a week to the Central Public Health Laboratory (Amman, Jordan). In Latin America, specimens were tested at each field site and then shipped to NAMRU SOUTH headquarters (Lima, Peru) for further molecular analysis.

### Laboratory Testing

In Latin America, NAMRU SOUTH tested all samples at field sites using the BioFire FilmArray System Respiratory Panel 2.1 (RP 2.1; bioMérieux, https://www.biomerieux.com), which detects 22 respiratory pathogens at 97.1% sensitivity and 99.3% specificity, including SARS-CoV-2. In Jordan, JMoH and NAMRU EURAFCENT used the Fast Track Diagnostics Respiratory Pathogens 33 (FTD-33; Siemens Healthineers, https://www.siemens-healthineers.com) assay, which has 76% sensitivity and 97% specificity (*7*). JMoH tested ILI cases only for influenza and SARS-CoV-2 during October 2020–January 2021. JCPHL tested influenza using singleplex CDC methods (*8*), then the FTD-33 assay. SARS-CoV-2 was tested using TaqPath COVID-19 CE-IVD RT-PCR Kit (Applied Biosystems, https://www.thermofisher.com). In Jordan and Latin America, results were reported daily or weekly to local ministry of health offices, public health authorities, and NAMRU SOUTH or NAMRU EURAFCENT.

### Data Analysis

We conducted a descriptive analysis for which we calculated 95% CIs using a binomial distribution. We used Stata version 16 software (StataCorp LLC, https://www.stata.com) for statistical analyses. We assessed frequency distribution of pathogens as number of specific pathogens over total number of common pathogens between FTD-33 and BioFire FilmArray RP 2.1 tests. We described trends of seasonality for influenza, SARS-CoV-2, RSV, and rhinovirus/enterovirus (RV/EV) as the monthly proportion of positive samples among total samples tested.

## Results

### Patient Demographics

A total of 11,305 samples were collected in Jordan (2,782 from ILI cases and 8,523 from SARI cases) and 4,841 in Latin America (4,154 from ILI cases and 687 from SARI cases). Most (50.1% in Jordan and 51.7% in Latin America) samples were collected during 2021 (Table 1). ILI cases were concentrated in the 16–50 year age group (58.7%) in both regions, in contrast to SARI cases, which were reported more (68.9%) in younger persons (<16-year age group) in Latin America, and more (44.7%) in older persons (>50-year age group) in Jordan (Tables 2, 3).

**Table 1:** Characteristics of samples collected as part of respiratory disease surveillance in the Middle East and Latin America during the COVID-19 pandemic, 2020–2022*

	Jordan				Latin America		
Characteristics	SARI, n = 8,523		ILI, n = 2,782		SARI, n = 687		ILI, n = 4,154
Year of enrollment							
2020	909 (11)		137 (5)		3 (0.4)		44 (1.1)
2021	4,408 (52)		1,251 (45)		252 (37)		2,250 (54)
2022	3,206 (38)		1,394 (50)		432 (63)		1,860 (45)
Total samples tested							
Influenza	8,523 (100)		2,782 (100)		687 (100)		4,154 (100)
SARS-CoV-2	8,144 (96)		2,782 (100)		687 (100)		4,154 (100)
Other respiratory pathogens	8,147 (96)		2,467 (89)		687 (100)		4,154 (100)
Detections							
No detections	4,446 (52.2)		1,655 (59.5)		116 (16.9)		1,088 (26.2)
Positive, % of positive samples	4,077 (47.8)		1,127 (40.5)		571 (83.1)		3,066 (73.8)
Monodetections	2,661 (65.3)		721 (64.0)		385 (67.4)		2,609 (85.1)
Codetections	1,416 (34.7)		406 (36.0)		186 (32.6)		457 (14.9)
2 pathogens	888 (21.8)		255 (22.6)		139 (24.3)		388 (12.7)
3 pathogens	341 (8.4)		104 (9.2)		36 (6.3)		56 (1.8)
>3 pathogens	187 (4.6)		47 (4.2)		11 (1.9)		13 (0.4)

*Samples were tested by Fast-Track Diagnostics 33 (FTD-33; Siemens Healthineers, https://www.siemens-healthineers.com) panel in Jordan and by BioFire FilmArray Respiratory Panel 2.1 (RP 2.1; bioMérieux, https://www.biomerieux.com) in Latin America. Values are no. (%). ILI, influenza-like illness; SARI, severe acute respiratory illness.

**Table 2:** Characteristics of ILI case-patients detected as part of respiratory disease surveillance in the Middle East and Latin America during the COVID-19 pandemic, 2020–2022*

Characteristic	No. case-patients (% [95% CI])	
	Jordan	Latin America
Total ILI cases	2,782	4,154
Sex		
M	1,281 (46 [43.3–48.7])	2,144 (51.6 [49.5–53.2])
F	1,501 (54 [51.3–56.7])	2,010 (48.4 [46.3–50.5])
Age group, y		
<16	715 (25.7 [22.5–28.9])	1,088 (26.2 [23.6–28.8])
16–50	1,634 (58.7 [56.3–61.1])	2,437 (58.7 [56.8–60.7])
>50	433 (15.6 [12.2–19.0])	629 (15.1 [12.3–17.9])
Positive test result		
Influenza	113 (4.1 [3.4–4.8])	410 (9.9 [7.0–12.8])
SARS-CoV-2	273 (9.8 [8.7–10.9])	1,146 (27.6 [25.0–30.2])
RSV, n = 2,467	34 (1.4 [0.9–0.19])	304 (7.3 [4.4–10.2])
Other respiratory pathogens, n = 2,467	1,326 (53.7 [51.7–55.7])	1,747 (42.1 [39.8–44.4])

*ILI, influenza-like illness; RSV, respiratory syncytial virus.

**Table 3:** Characteristics of SARI case-patients detected as part of respiratory disease surveillance in the Middle East and Latin America during the COVID-19 pandemic, 2020–2022*

	Jordan		Latin America
Characteristic	No. case-patients (% [95% CI])		No. case-patients (% [95% CI])
Total SARI cases	8,523		687
Sex			
M	4,593 (53.9 [52.5–55.3])		368 (53.6 [48.5–58.7])
F	3,930 (46.1 [44.7–47.6])		319 (46.4 [41.5–51.8])
Age group			
<16	2,931 (34.4 [32.7–36.1])		473 (68.9 [64.7–73.0])
16–50	1,780 (20.9 [19.0–22.8])		123 (17.9 [11.1–24.7])
>50	3,812 (44.7 [43.1–46.3])		91 (13.2 [6.3–20.1])
Positive test result			
Influenza, n = 8,523	219 (2.6 [2.3–2.9])		52 (7.6 [4.0–14.8])
SARS-CoV-2, n = 8,144	1,361 (16.7 [15.9–17.5])		79 (11.5 [4.5–18.5])
RSV, n = 8,147	448 (5.5 [5.0–6.0])		134 (19.5 [12.8–26.2])
Other respiratory pathogens, n = 8,147	4,255 (52.2 [51.1–53.3])		556 (80.9 [77.6–84.2])

*ILI, influenza-like illness; RSV, respiratory syncytial virus; SARI, severe acute respiratory infection.

### Circulating Pathogens

**Figure 1:**
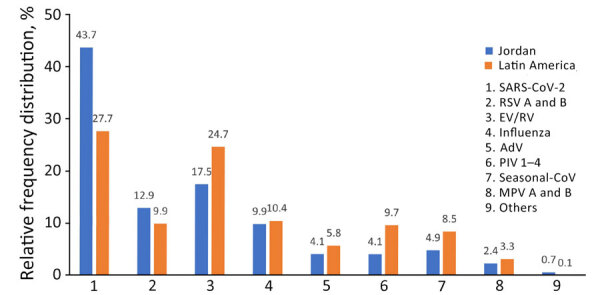
Overall relative frequency distribution of respiratory pathogens commonly tested as part of respiratory disease surveillance in the Middle East (Jordan) and Latin America during the COVID-19 pandemic, 2020–2022. Percentages were calculated relative to the total number of common targets tested by both Fast-Track Diagnostics 33 (FTD-33; Siemens Healthineers, https://www.siemens-healthineers.com) and BioFire FilmArray Respiratory Panel 2.1 (RP 2.1; bioMérieux, https://www.biomerieux.com) assays. Samples include 3,738 from Jordan and 4,4,28 from Latin America. Others includes *Chlamydia pneumoniae* (0.44% in Jordan and 0.07% in Latin America), *Bordetella pertussis* (0.1% in Jordan and 0.05% in Latin America), and *Mycoplasma pneumoniae* (0.17% in Jordan and 0.02% in Latin America). AdV, adenovirus; MPV, metapneumovirus; PIV, parainfluenza virus; RSV, respiratory syncytial virus; RV/EV, rhinovirus/enterovirus.

Of 11,305 tested samples from Jordan, 46% (5,204) were positive for >1 respiratory pathogen; of the 4,841 samples from Latin America, 75.1% (3,637) were positive. Pathogen positivity was higher in Latin America for both SARI (83.1%) and ILI surveillance (73.8%) than in Jordan (SARI 47.8% and ILI 40.5%). The rate of monodetection, the detection of a single pathogen, in SARI was similar in both regions, 65.3% in Latin America and 67.4% in Jordan; in contrast, monodetections among ILI cases were significantly higher (85.1%) in Latin America than in Jordan (64.0%) (Table 1). In addition, a total of 4,428 pathogens were identified in Latin America and 7,994 in Jordan, of which 3,738 pathogens were common targets identified by the tests performed. Frequency distribution of those pathogens tested in both regions showed that SARS-CoV-2 was the most recurrently detected respiratory pathogen in both regions, 43.7% in Jordan and 27.7% in Latin America; RV/EV was next most detected at 17.5% in Jordan and 24.7% in Latin America (Figure 1).

### Patterns of Seasonality

**Figure 2:**
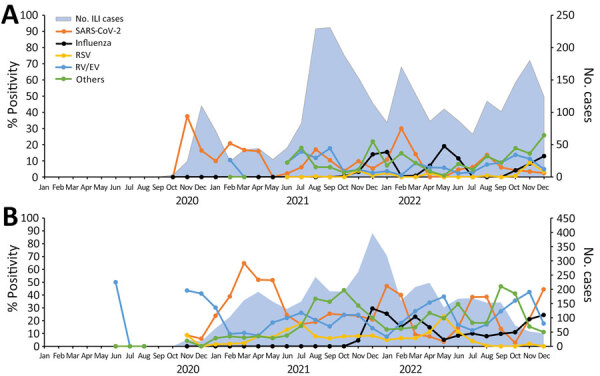
Percent positivity of different respiratory pathogens associated with ILI in detected as part of respiratory disease surveillance in the Middle East (Jordan, A) and Latin America (B) during the COVID-19 pandemic, 2020–2022. In Jordan, testing ILI cases with Fast-Track Diagnostics 33 (FTD-33; Siemens Healthineers, https://www.siemens-healthineers.com) started in February 2021. During October 2020–January 2021, ILI cases were tested only for influenza and SARS- CoV-2. Others includes *Chlamydia pneumoniae*, *Bordetella pertussis*, and *Mycoplasma pneumoniae*. ILI, influenza-like illness; RSV, respiratory syncytial virus; RV/EV, rhinovirus/enterovirus.

**Figure 3:**
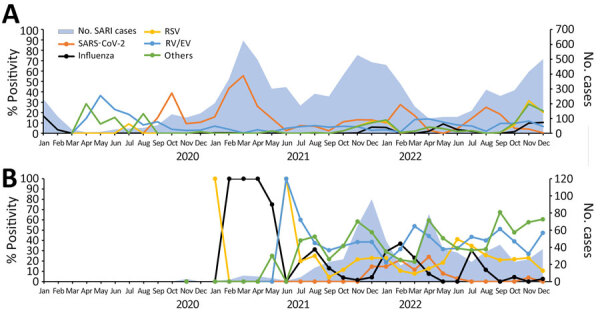
Percent positivity of different respiratory pathogens associated with SARI detected as part of respiratory disease surveillance in the Middle East (Jordan, A) and Latin America (B) during the COVID-19 pandemic, 2020–2022. Others includes *Chlamydia pneumoniae*, *Bordetella pertussis*, and *Mycoplasma pneumoniae*. ILI, influenza-like illness; RSV, respiratory syncytial virus; RV/EV, rhinovirus/enterovirus; SARI, severe acute respiratory infection.

During the surveillance period, 4 peaks of SARS-CoV-2 were reported in Jordan (September 2020, March 2021, February 2022, and August 2022) and 3 peaks in Latin America (March 2021, February 2022, and July 2022). Temporal pathogen distribution by region showed that influenza virus was not detected until October 2021 in Jordan and November 2021 in Latin America. During the influenza silent period, March 2020–November 2021, pathogens, including SARS-CoV-2, RSV, and RV/EV continued to circulate (Figures 2, 3).

## Discussion

Globally, circulation of respiratory viruses was disrupted during the COVID-19 pandemic; the magnitude, timing, and duration of the disruption varied across viruses and geographic locations. We found that even though Jordan used an extended panel that detected 33 pathogens in addition to PCR for influenza and SARS-CoV-2, the positivity rates were much higher for both SARI (83% vs. 48%) and ILI (74% vs. 41%) in Latin America, which used FilmArray RP 2.1 to detect 22 pathogens including SARS-CoV-2 and influenza. Those findings could be explained by the case definitions used in each region. In Jordan, WHO ILI and SARI case definitions require fever and cough within the past 10 days in addition to hospitalization for SARI cases. In contrast, the modified WHO case definition used in Latin America included sore throat or rhinorrhea but limited fever onset to within the past 48 hours.

Influenza virus was not detected until October 2021 in Jordan and November 2021 in Latin America, as reported (*9*,*10*). In Jordan, influenza peak was detected during its usual pattern in December–January. In Latin America, however, influenza began to peak in November 2021, which was out of season for nontropical Latin America, where it typically peaks during June–August (*11*), and Central America, where it usually is present year-round (*12*). Influenza detection decreased globally but other noninfluenza pathogens, including SARS-CoV-2, were detected despite strict physical preventive measures and reductions in travel; that trend suggests different or more efficient transmission mechanisms or pathogen survival on surfaces (*13*).

Unlike influenza, SARS-CoV-2 peaks were observed in January–February 2022 in both regions, which could have been from the combination of the emergence of the Omicron variant and the global relaxing of preventive measures (*14*). In addition, ILI surveillance in Latin America revealed that during periods of decreased detection of SARS-CoV-2, the number of non–SARS-CoV-2—positive cases increased, suggesting a depletion of susceptible populations for SARS-CoV-2 and opportunities for non–SARS-CoV-2 pathogens to circulate. In Jordan, SARI data showed that during periods of intensive SARS-CoV-2 prevention measures, detection of other respiratory pathogens decreased (Figure 3). Like influenza virus and RSV, circulation of other respiratory pathogens, including seasonal human coronavirus, human parainfluenza virus, and human metapneumovirus, was notably lower at the onset of the COVID-19 pandemic when preventive measures were in place.

In Jordan during the 4 peaks of COVID-19, positivity of the other respiratory pathogens in SARI cases decreased (Figure 3), most likely because of the measures implemented in response to the increase in COVID-19 cases. Our observation (data not shown) is consistent with other studies conducted in different regions of the world showing a significant decline in respiratory virus circulation during the COVID-19 pandemic as increased preventive measures were undertaken (Figures 2, 3).

For RSV, sporadic cases were detected in Jordan during July and December 2020, and in May and August 2021, which is different than the winter season (December–February) peak typically seen in Jordan (*15*). Increased activity of RSV in Latin America was reported in June–July 2021 and May–June 2022; with similar increases were reported in the United States later in October–November 2022 (*16*). 

Although Latin America and Jordan surveillance sites were in different hemispheres, we observed a similar temporal pattern of SARS-CoV-2. Those similarities may have been driven by the introduction of new SARS-CoV-2 variants rather than seasonal factors (*17*).

We describe the results of surveillance systems in 2 different geographic locations that continued to function during the COVID-19 pandemic, despite the public health challenges during the global emergency. Moreover, expanding testing beyond SARS-CoV-2 and influenza viruses highlighted the role of other respiratory pathogens that add to the burden of the ILI and SARI and potential interactions that affect the epidemiology of respiratory infections.

Because we relied on passive surveillance, our study population was limited to patients seeking medical care at health centers, which may have changed during the pandemic. It is possible that, during COVID-19 waves, hospitalization criteria required for SARI changed. Those variables could explain why fewer other respiratory viruses were detected during COVID-19 waves. In addition, Latin America surveillance sites are predominantly for ILI surveillance; in Jordan, SARI cases were higher priority. Finally, our study did not address how differences in laboratory techniques and case definitions affected detection of respiratory pathogens.

In conclusion, our study showed that respiratory pathogens other than SARS-CoV-2 were circulating during the COVID-19 pandemic in 2020–2022. The expanded use of multiplex molecular respiratory virus assays and the increased awareness of the public health burden of respiratory pathogens have renewed interest in characterizing the epidemiology of respiratory viruses. As demonstrated during the COVID-19 pandemic, continued sentinel surveillance is vital for assessing the burden of respiratory diseases globally.

Appendix Additional information on respiratory disease surveillance in the Middle East and Latin America during the COVID-19 pandemic, 2020–2022.
