# Supplementary material for: Respiratory Disease Surveillance in the Middle East and Latin America during the COVID-19 Pandemic, 2020–2022
Source: Emerg Infect Dis. 2024 Nov;30(Suppl 2):S26–32. doi: 10.3201/eid3014.240303 (PMC11559572; doi:10.3201/eid3014.240303)
Supplement: Appendix — Additional information on respiratory disease surveillance in the Middle East and Latin America during the COVID-19 pandemic, 2020–2022. [file 24-0303-Techapp-s1.pdf]

Article DOI: https://doi.org/10.3201/eid3014.240303

EID cannot ensure accessibility for supplementary materials supplied by authors. Readers who have difficulty accessing supplementary content should contact the authors for assistance.

Respiratory Disease Surveillance in the Middle East and Latin America during the COVID-19 Pandemic, 2020–2022

Appendix

Appendix Table 1. Sites names by target population and region in Jordan and six Latin American countries, 2020–2022

ILI sites	SARI sites
Jordan	
1- Amman AlShamel primary healthcare center located in Amman Governorate (the capital)	1- Prince Hamza Hospital located in Amman Governorate center of Jordan
2- Alsareeh primary healthcare center located in Irbid Governorate, north of Jordan	2- King Abdullah University Hospital located in Irbid Governorate, north of Jordan
3- Almazar primary healthcare center located in Karak Governorate, South of Jordan	3- AlKark Hospital located in Karak Governorate, South of the kingdom
4- Jarash primary healthcare center located in Jerash Governorate, mid north between Amman and Irbid	4- AlZarqaa Hospital in Alzarqaa Governorate, northeast of Amman the capital
Guatemala	
1- FUNSALUD, primary health center, Caballo Blanco	N/A
Panama	
1- Meteti primary health located in the Darien region	N/A
Honduras	
1- MEDEL primary health center, JTF-Bravo, Comayagua	N/A
Colombia	
1- Hospital Pablo Tobon Uribe, Medellin	1- Hospital Pablo Tobon Uribe
2- Secretaria de Salud del Meta, Villavicencio	2- Secretaria de Salud del Meta
3- C.S. La Esperanza, Cartagena	
4- CAP. Blas de Lezo, Cartagena	
5- CAP. Canapote, Cartagena	
6- CAP. Nuevo Bosque, Cartagena	
7- CAP. Olaya Herrera, Cartagena	
Paraguay	
1- Hospital Regional de Boqueron, Mariscal Estigarribia	1- Hospital Regional de Boqueron
Peru	
	1- Hosp. Apoyo Iquitos, Loreto
1- C.S. Zarumilla, Tumbes	2- Hosp. Edgardo Rebagliati, Lima
2- C.S. Bagua, Amazonas	3- Hosp. Militar Central, Lima
3- C.S. Bellavista-Nanay, Loreto	4- Hosp. Regional Demarini Caro, Junin
4- C.S. Quistococha, Loreto	5- Hosp. Regional FAP, Loreto
5- C.S. Zungarococha, Loreto	6- Hosp. Regional Loreto, Loreto
6- C.S. Jorge Chavez, Madre de Dios	7- Hosp. Santa Gema, Loreto,
7- C.S. La Joya, Madre de Dios	
8- C.S. Nuevo Milenio, Madre de Dios	
9- C.S. Pachitea, Piura	
10- C.S. San Jeronimo, Cusco	
11- Spanish Schools, Cusco (Western Travelers)	
12- Hosp. Apoyo Iquitos, Loreto	
13- Hosp. Regional Julio C.Demarini Caro, Junin	
14- Hosp. Regional Loreto, Loreto	
15- Hosp. Santa Gema, Loreto	
16- Clinica Naval, Iquitos (Navy), Loreto	

ILI sites	SARI sites
17- C.S. Militar (Army), Trujillo, La Libertad	
18- Hosp. Militar Santa Rosa (Army), Loreto	
19- Hosp. Regional FAP (Air Force), Loreto	
20- NAMRU SOUTH, Lima	
21- NAMRU SOUTH, Iquitos, Loreto	

Appendix Table 2. ILI and SARI case definitions used in in Jordan and six Latin American countries, 2020–2022

Country	ILI	SARI
Kingdom of Jordan	WHO case definition	
	An acute respiratory infection with:<br>• measured fever of 38 °C and cough<br>• with onset within the last 10 days	An acute respiratory infection with:<br>• history of fever or measured fever of ≥ 38 C°.<br>• and cough.<br>• with onset within the last 10 days.<br>• and requires hospitalization
Latin America	WHO modified case definition	
	An acute respiratory infection with:<br>• measured fever of 38°C in the last 48hrs<br>• and cough or sore throat or rhinorrhea<br>• with onset within the last 5 days	An acute respiratory infection with:<br>• history of fever or measured fever of 38°C;<br>• cough or sore throat or rhinorrhea or<br>• myalgia, with onset within the last 10 days and require hospitalization
	Peruvian MoH COVID-19 case definition 2020-2022	
	An acute respiratory infection with onset within the last 10 days<br>with 2 or more of the following symptoms:<br>• cough or sore throat or myalgia or<br>• stuffy nose or fever	An acute respiratory infection with onset within the last 10 days<br>with 2 or more of the following symptoms:<br>• cough or sore throat or myalgia or<br>• stuffy nose or fever<br>• and requires hospitalization

Appendix Table 3. Respiratory pathogens tested by FTD-33 and Biofire RP 2.1. Tests used in in Jordan and six Latin America countries, 2020–2022.

Virus	FTD-33	RP 2.1
SARS-CoV-2	N	Y
Influenza virus		
influenza A	Y	Y
influenza A (H1N1) pdm09	Y	Y
influenza A (H1N1)	N	Y
influenza A (H3N2)	Y	Y
influenza B	Y	Y
influenza C	Y	N
Parainfluenza virus (PIV)		
PIV1	Y	Y
PIV2	Y	Y
PIV3	Y	Y
PIV4	Y	Y
Seasonal Coronavirus (CoV)		
CoV NL63	Y	Y
CoV 229E,	Y	Y
CoV OC43,	Y	Y
CoV HKU1	Y	Y
Human Metapneumovirus (hMPV) A&B	Y	Y
Rhinovirus/Enterovirus (RV/EV)	N	Y
Rhinovirus	Y	N
Enterovirus	Y	N
Respiratory Syncytial Virus (RSV)A &B	Y	Y
Adenovirus	Y	Y
Parechovirus	Y	N
Bocavirus	Y	N
**Bacteria**		
Pneumocystis jirovecii	Y	N
Mycoplasma pneumoniae	Y	Y
Chlamydia pneumoniae	Y	Y
Streptococcus pneumoniae,	Y	N

Virus	FTD-33	RP 2.1
Hemophilus influenzae	Y	N
Hemophilus influenzae type B	Y	N
Staphylococcus aureus	Y	N
Moraxella catarrhalis	Y	N
Bordetella species (excluding *B. parapertussis*)		
Bordetella pertussis	Y	Y
Bordetella parapertussis	N	Y
Klebsiella pneumoniae	Y	N
Legionella sp	Y	N
Salmonella sp	Y	N
